# Microwave-Assisted Green Synthesis of Highly Crystalline Copper-Doped ZnO Nanoparticles: Enhanced Antimicrobial and Antifibrotic Effects for Wound Healing

**DOI:** 10.7759/cureus.97191

**Published:** 2025-11-18

**Authors:** Shubha P., Harini K. S., Shyamsundar S., Preethi Kusugal

**Affiliations:** 1 Biomaterials Research and Development, Subbaiah Research Institute, Shimoga, IND; 2 Prosthodontics, Crown, Bridge, and Implantology, Subbaiah Institute of Dental Sciences, Shimoga, IND; 3 Oral and Maxillofacial Surgery, JSS Dental College and Hospital, JSS Academy of Higher Education and Research, Mysore, IND; 4 Prosthodontics, Crown, and Bridge, Maratha Mandals Dental College, Belagavi, IND

**Keywords:** antifibrotic, antimicrobial, cu-zno, doping, fibroblasts, mmp, nanoparticles

## Abstract

Introduction: Wound healing involves controlled proliferation of fibroblasts and synthesis of extracellular matrix (ECM). Imbalance in this process leads to excessive fibroblast activity, collagen deposition, and fibrosis. Current pharmacological therapies target the inhibition of profibrotic signaling pathways, and surgical procedures are reserved for advanced pathologic conditions, necessitating research on local therapies. The present study focuses on investigating the antifibrotic potential of Cu-doped ZnO (Cu-ZnO) nanoparticles (NPs) synthesized using a microwave-assisted green synthesis method.

Methods: ZnO and Cu-ZnO NPs were synthesized utilizing microwave energy using *O sanctum* hydroalcoholic extract. Synthesized NPs were characterized using appropriate characterization techniques to elicit the crystallinity, size, morphology, and optical properties. Using the disk diffusion assay, antimicrobial activity of NPs was determined against *S aureus,* and hematocompatibility was evaluated using chick blood. Inhibition of fibroblastic activity and mitochondrial membrane potential (MMP) was determined to explore antifibrotic activity.

Results: X-ray diffraction analysis confirmed successful Cu²⁺ incorporation into the ZnO lattice, with distinct (200) and enhanced (101) diffraction peaks and a reduction in crystallite size at higher dopant levels. Morphological assessment revealed a transition from irregularly shaped ZnO NPs to well-defined tripod-like Cu-ZnO nanoforms, indicating directional growth. UV-Visible (UV-Vis) spectroscopy showed a red shift, reflecting the narrowing of the band gap upon Cu doping. Antimicrobial evaluation against* Staphylococcus aureus* demonstrated a dopant- and dose-dependent enhancement in activity, with the bacterial zone of inhibition (ZOI) increasing from 8.5 mm (undoped ZnO) to 14 mm (3 M Cu-ZnO) at the highest tested concentration of 1.0 mg/mL, and measurable inhibition (5 mm) persisting even at 0.25 mg/mL for 3 M Cu-ZnO NPs. Hemolysis remained below 5% for all formulations, confirming hemocompatibility. As per the results of antifibrotic activity assays, 3 M Cu-ZnO NPs showed the strongest suppression of 3T3 fibroblast proliferation, with JC-1 analysis confirming mitochondrial membrane depolarization leading to the apoptosis of fibroblasts due to excessive ROS production by Cu-ZnO NPs.

Conclusions: Cu-ZnO NPs exhibited strong antimicrobial activity, maintained hemocompatibility, and effectively regulated the proliferation of the fibroblasts. The enhanced antifibrotic effect is mainly driven by elevated ROS levels, which induce mitochondrial membrane depolarization, as confirmed by the JC-1 assay, leading to mitochondrial stress and apoptotic elimination of hyperproliferative fibroblasts.

## Introduction

Wound healing is a complex and dynamic process supported by a multitude of cellular events [1] that need to be strictly coordinated to repair damaged tissue efficiently. The normal wound healing process involves a cascade of events that involves coordinated interplay of cells, growth factors, and extracellular matrix (ECM) to achieve scar-free repair [2]. When this delicate balance between inflammation, proliferation, and remodeling gets disrupted, an aberrant wound healing takes place, which is manifested as visible scars (keloid and hypertrophic scars) to internal organ fibrosis, and chronic non-healing wounds [3]. The excessive scarring is characterized by disorganized and redundant deposition of ECM resulting from abnormal proliferation and differentiation of fibroblasts; it has many adverse consequences, including disfiguring, pain, itching, contracture, and locomotive restriction, inflicting the injured both physically and psychologically, necessitating targeted therapeutic interventions [4].

Hence, the therapeutic intervention should be directed towards regulating fibroblastic activity and preventing uncontrolled ECM accumulation to achieve balanced wound repair and functional tissue regeneration. Current antifibrotic therapies include pharmacological and surgical approaches aimed at limiting excessive fibroblast activity and collagen deposition. Pharmacological agents like perfenidone, nintedanib, and imatinib, which inhibit profibrotic pathways, are often used in antifibrotic therapy. Corticosteroids, methotrexate, and antioxidants modulate inflammation and oxidative stress [5]. Surgical options include scar revision, organ transplantation, and laser or microneedling to address severe or established fibrosis [6]. However, these methods have limitations in efficacy or safety, highlighting the need for novel therapies that are locally active and capable of modulating fibroblast behavior to promote scar-free healing.

Metal oxide nanoparticles (NPs) are promising in regenerative medicine due to their tunable physico-mechanical and biological properties [7]. Among these, zinc oxide (ZnO) NPs are particularly attractive owing to their tunable band gap and established biocompatibility. ZnO is known to stimulate keratinocyte migration, enhance angiogenesis, and accelerate wound re-epithelialization, with additional antimicrobial benefits [8]. However, the high bandgap energy and rapid electron-hole recombination limit their biological efficacy.

To overcome these limitations, doping and heterojunction formation have been explored as strategies to enhance the functionality of ZnO [9]. Among various metallic dopants, copper is one of the most effective, particularly for enhancing properties relevant to biomedical applications of ZnO [10].

Cu ions introduce defect states within the ZnO lattice, narrowing the bandgap and facilitating visible light-driven reactive oxygen species (ROS) generation [11]. These ROS species can modulate fibroblast proliferation, induce mild oxidative signaling, and suppress fibrotic responses at controlled concentrations [12]. Additionally, Cu plays an essential physiological role in angiogenesis, collagen cross-linking, and tissue remodeling, which are the key processes in wound repair [13].

Considering this, in the present work, Cu-ZnO NPs were green-synthesized using *Ocimum sanctum* hydroalcoholic extract through a microwave-assisted method, wherein the plant phytochemicals acted as natural reducing and capping agents. It was hypothesized that Cu incorporation into ZnO would enhance their biological functionality by enabling controlled modulation of fibroblast behavior, promoting wound repair, and suppressing excessive ECM deposition. Accordingly, the objective of the present work was to synthesize and characterize Cu-ZnO NPs and evaluate their antifibrotic, wound-healing, hemocompatible, and antimicrobial properties to establish their potential as a dual-action, scar-regenerative wound therapeutic platform.

## Materials and methods

Microwave-assisted green synthesis of Cu-ZnO and undoped ZnO NPs

Microwave-assisted green synthesis method was used in the fabrication of Cu-ZnO NPs. Undoped ZnO NPs were also synthesized to establish a baseline.

Zn(NO₃)₂.6H_2_O (Alfa Aesar, Haverhill, MA) as a source of Zn ions; Cu(NO₃)₂ (SD Fine Chemicals Pvt. Ltd., Mumbai, India); NaOH (Fisher Scientific, Waltham, MA); ultrapure water of resistivity 18.2 MΩ (Purelab® Option-Q, ELGA LabWater, High Wycombe, UK) were used in the synthesis of Cu-ZnO and undoped ZnO NPs. *O sanctum* hydroalcoholic extract bioreductant was synthesized using our previous protocol [14].

**Figure 1:**
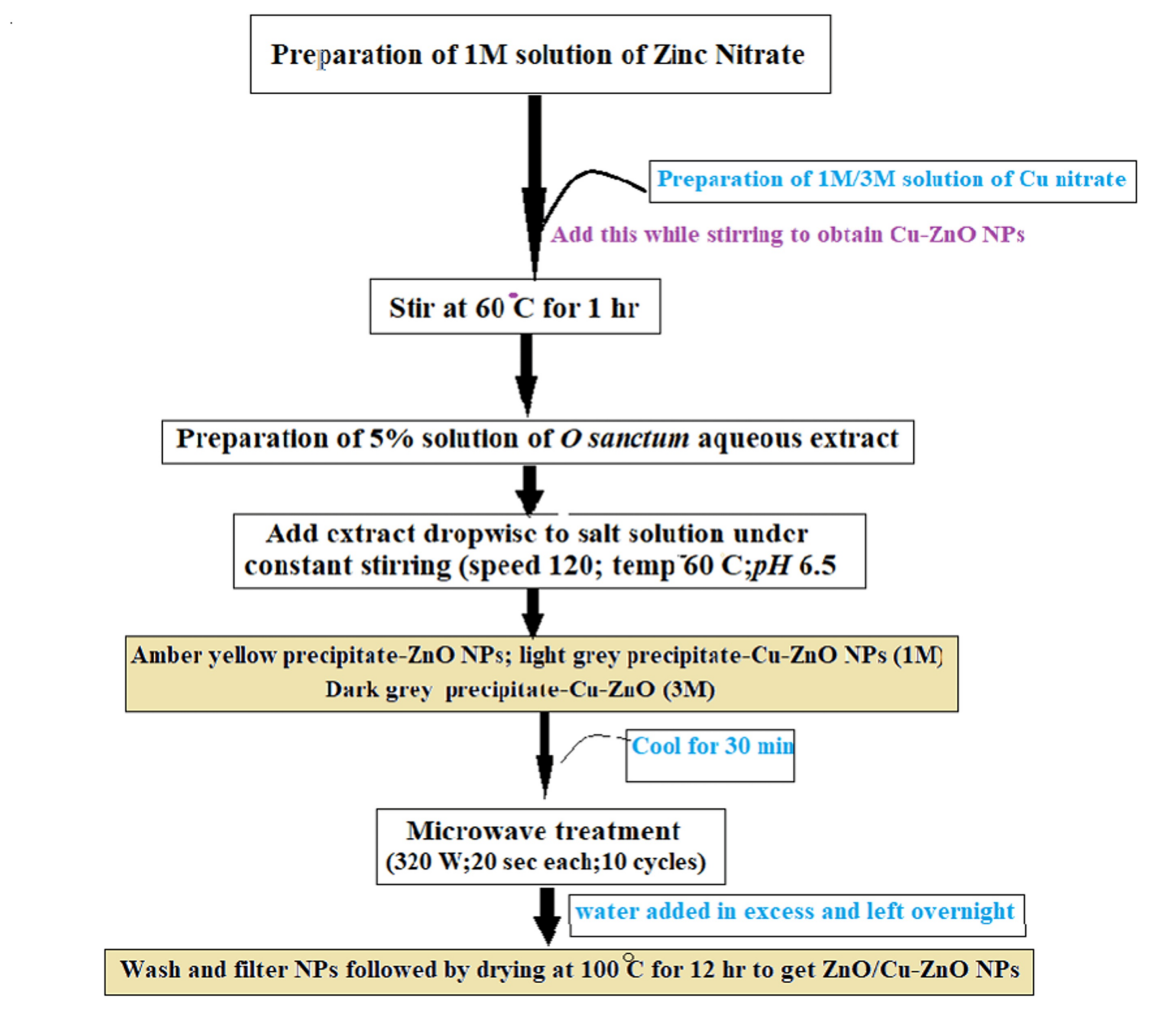
Flowchart showing the scheme of synthesis of ZnO and Cu-ZnO NPs (1 and 3 M). Image credit: Shubha P.

To synthesize ZnO NPs, a 1 M solution of Zn(NO₃)₂.6H_2_O was prepared by dissolving 14.87 g of salt in 50 mL ultrapure water and maintaining at 60 °C for 1 hour with continuous stirring. 50 ml of 5% hydroalcoholic extract of *O sanctum *was dissolved in ultrapure water and added to the precursor solution dropwise with constant stirring. Formation of yellowish brown precipitate was observed. Following a bench cooling session of 30 minutes, the solution with precipitate was transferred to a 250 mL capacity microwave-safe bottle and microwave-treated for 2 minutes (20 seconds/cycle; total 10 cycles) at 320 Watts. The color of the mixture changed from yellowish brown to dark brown. Ultrapure water in excess was added to this and cooled overnight. Further, the NPs were washed several times and dried at 100 °C for 12 hours. Figure 1 shows the synthesis scheme.

**Figure 2:**
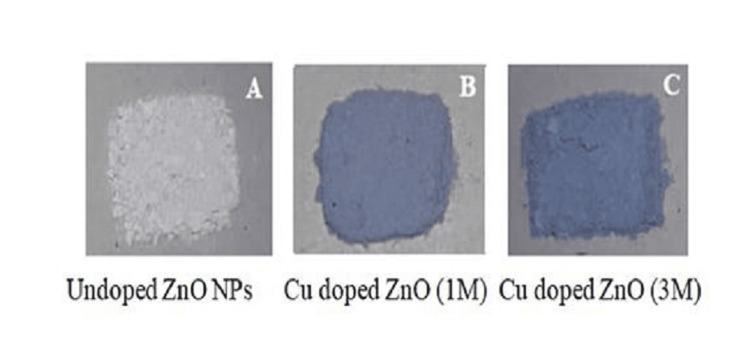
(A) Pure ZnO NPs; (B) 1 M Cu-ZnO NPs; (C) 3 M Cu-ZnO NPs. NP, nanoparticle

Cu-ZnO NPs were also synthesized using the same procedure with two different stoichiometries of Cu salt. Fifty milliliters of a 1 M solution of Cu(NO₃)₂ (0.36 g in 50 mL ultrapure water) was prepared and maintained separately at 30 °C. Similarly 3 M solution of Cu(NO₃)₂ was obtained by adding 1.08 g of Cu salt to 50 mL of ultrapure water. This was further added to Zn(NO₃)₂.6H_2_O, under constant stirring. Further steps were carried out, like the synthesis of undoped ZnO NPs as mentioned above, and a grayish-colored fine powder of Cu-ZnO NPs was obtained after drying at 100 °C (Figure 2).

Characterization 

The physical and morphological characterization of Cu-ZnO and ZnO NPs was performed at the Central Instrumentation Facility, Vijnan Bhavan, University of Mysore. X-ray diffraction studies (PXRD) (Rigaku SmartLab II, Rigaku Corporation, Tokyo, Japan) confirmed crystal structure; dynamic light scattering (DLS) (Microtrac Zeta Analyzer, Microtrac MRB, York, PA) determined particle size; UV-Visible (UV-Vis) spectroscopy (Elico SA-165, Elico Ltd., Hyderabad, India) analyzed optical properties; and scanning electron microscopy (SEM) (Hitachi S-3400N, Hitachi High-Tech Corporation, Tokyo, Japan) examined morphology and elemental composition.

Determination of antimicrobial activity of ZnO and Cu-ZnO NPs against *S. aureus* using disk-diffusion assay

The antibacterial activity of Cu-ZnO (1 and 3 M) and ZnO NPs was carried out against *S. aureus* using the disk diffusion method. Respective NPs were serially diluted in the concentration range 1.0-0.125 mg/mL. Pure cultures of *S. aureus* (MTCC 5908) were prepared in Luria broth (HIMEDIA) with final concentration adjusted to 1x10^6 ^CFU/mL using 0.5 McFarland standard. Filter paper disks measuring 5 mm in diameter were prepared using Whatman’s filter paper and were UV sterilized. Thirty microliters of fresh inoculum was spread on the agar surface using a sterile L-rod. The filter paper disks were saturated with the NPs suspension for 30 seconds. The disks were carefully picked and placed on the agar surface. Sterile ultrapure water was used as a control. The petri dishes were incubated at 37 °C, and results were observed after 24-48 hours.

In vitro hemotoxicity of synthesized NPs using the hemolysis assay

Hemolysis assay is mainly performed to study the lysis of RBC with respect to the change in isotonicity of the solution in the presence of the test substance. Using Halfman’s group standard procedure, in vitro hemolysis caused by various concentrations of ZnO and Cu-ZnO NPs was carried out using the fresh chick blood [14]. To 20 mL of fresh blood, 1 mL of anticoagulant was added, and washed thrice with saline using an ultra-centrifuge at 2,000 rpm for 5 minutes (Centrifuge 5430R, Eppendorf, Hamburg, Germany) and dispersed in 1× Dulbecco’s phosphate-buffered saline (DPBS).

About 2 mg of ZnO and Cu-ZnO NPs were dispersed in 2 mL ultrapure water and sonicated for 45 minutes to obtain complete dispersion and serially diluted up to 0.125 mg/mL. Blood stock solution was prepared in DPBS such that the mixture contains ~5 × 10^8^ RBC/mL. This was exposed to serially diluted samples of NPs and incubated for 3 hours. Further, the contents were centrifuged, and the supernatant was collected. Absorbance was read at 550 nm (for ZnO) and 388 nm (for Cu-ZnO) using 1× DPBS as a reference. Hemolysis caused by various groups of NPs was calculated using the formula:

\[ \frac{AB_s - AB_n}{AB_p - AB_n} \]

where ABs is the absorbance of the sample, ABn is the absorbance of the negative control, and ABp is the absorbance of the positive control.

Assessment of antifibrotic activity of ZnO and Cu-ZnO NPs using cell proliferation assay and determination of mitochondrial membrane potential

The antifibrotic potential of Cu-ZnO (1 and 3 M) and ZnO NPs was evaluated using the WST-8 assay in Balb/3T3 mouse fibroblast cell lines (National Center for Cell Science, Pune). The cells were cultured in Dulbecco’s Modified Eagle Medium (DMEM) supplemented with 10% fetal bovine serum (FBS) and antibiotics (50 μg/mL penicillin and streptomycin) at 37 °C in a humidified 5% CO₂ atmosphere. NPs suspensions (1-0.125 mg/mL) were serially diluted in sterile PBS, ultrasonicated for 45 minutes for uniform dispersion, and 10 μL of each was added to cultured cells. After 24-hour incubation, 10 μL of CCK-8 reagent was added, and plates were incubated for 4 hours [15]. Absorbance was recorded at 450 nm using a microplate reader, and percentage inhibition was calculated. To further assess mitochondrial integrity, mitochondrial membrane potential (ΔΨm) was evaluated using the JC-1 dye assay. After 24-hour NPs exposure, cells were stained with JC-1 (5 µg/mL) for 20 minutes at 37 °C, washed with PBS, and fluorescence was recorded at 485/530 nm (green). The intensity of green fluorescence was used as an indicator of mitochondrial depolarization [16].

Statistical analysis 

The antimicrobial activity, hemolytic activity, and antifibrotic activity were carried out in triplicate. The average value was calculated using SPSS software version 30 (IBM Corp., Armonk, NY) and reported accordingly. 

## Results

Results of characterization 

X-ray Diffraction Studies 

**Figure 3:**
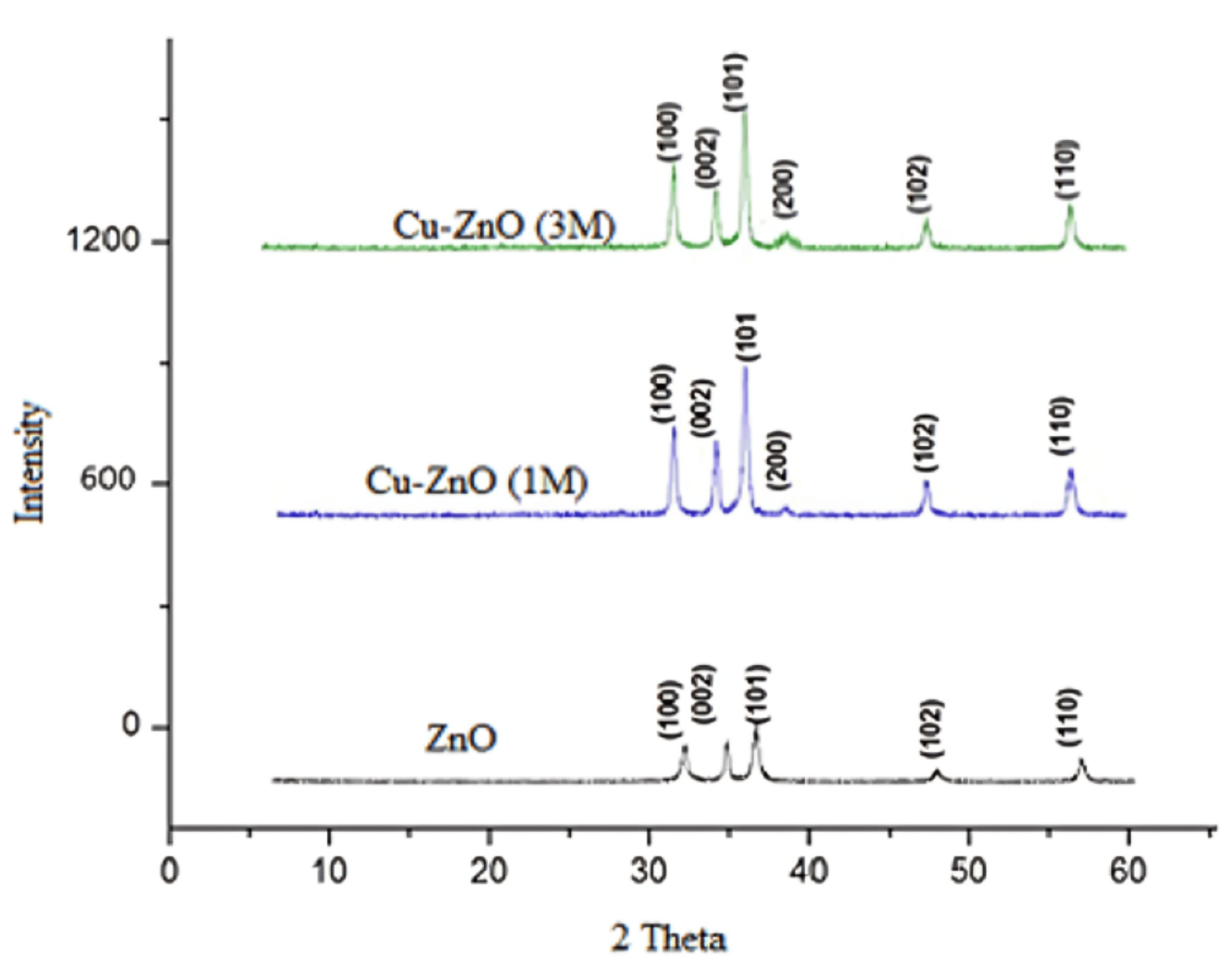
Powder X-ray diffractogram of ZnO and Cu-ZnO NPs. NP, nanoparticle

XRD analysis of green-synthesized ZnO and Cu-ZnO NPs confirmed a hexagonal wurtzite structure with characteristic peaks at 2θ values corresponding to JCPDS 36-1451. Variation in peak intensity and the emergence of a new peak at (200) plane indicated successful Cu incorporation into the ZnO lattice (Figure 3). Increased Cu concentration (1 to 3 M) enhanced peak intensity at (101) and (200), suggesting Cu-ZnO cluster formation and partial phase segregation [17].

FTIR Spectroscopy

**Figure 4:**
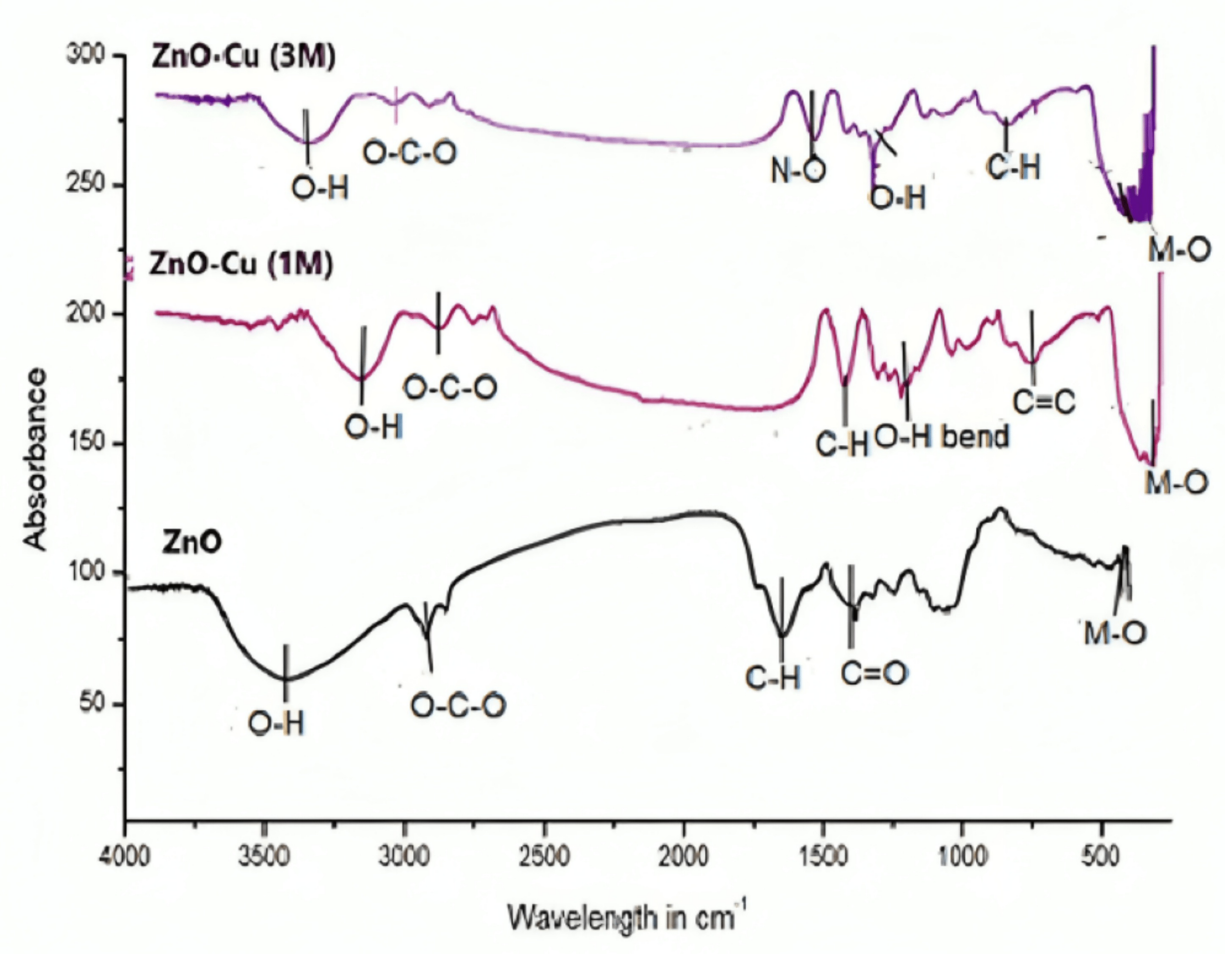
FTIR spectra of ZnO and Cu-ZnO with major functional groups. FTIR, Fourier-transform infrared

Fourier-transform infrared (FTIR) spectra (Figure 4) and the corresponding band assignments (Table 1) of pure and Cu-ZnO NPs reveal peaks corresponding to aromatic, carboxylic, and hydroxyl groups, indicating the presence of phytochemical residues from *O. sanctum* in the synthesized NPs.

**Table 1:** Major FTIR functional groups of ZnO and Cu-ZnO NPs. FTIR, Fourier-transform infrared; NP, nanoparticle

Band (cm^-1^)	Assignment (ZnO)	Assignment (1 M Cu-ZnO)	Assignment (3 M Cu-ZnO)
3195-3775	-OH stretch (alcohol)	-OH stretch (water)	–OH stretch (water)
2460-2399	O-C-O Stretch (CO_2_)	O-C-O stretch (CO_2_)	O-C-O stretch
1667	Weak –CH bend due to aromatic group	Weak –CH bend due to aromatic group	-
1867	C=O due to weak anhydride	-	-
1543	-	-	N-O_2_ stretch
1434	-	-OH bend due to carboxylic acid	
1385	-	-	-CH bend medium due to the aldehyde group
769	-	-	1,2-disubstituted –CH bend
542-564	Peak corresponding to Zn-O formation	Zn-O(M-O), possible Cu doping)	Zn-O(M-O) (possible Cu doping)

ZnO exhibited a characteristic absorption band near 550 cm⁻¹, which shifted to higher frequencies (550.01 cm⁻¹ for 1 M Cu-ZnO and 564.98 cm⁻¹ for 3 M Cu-ZnO) upon Cu incorporation, attributed to substitutional effects of the lighter Cu atoms [17]. Additional peaks corresponding to -OH, C-H, and N-O vibrations indicate the presence of alcoholic, carboxylic, and amine groups from the *O. sanctum* extract.

UV-Vis Spectroscopy

**Figure 5:**
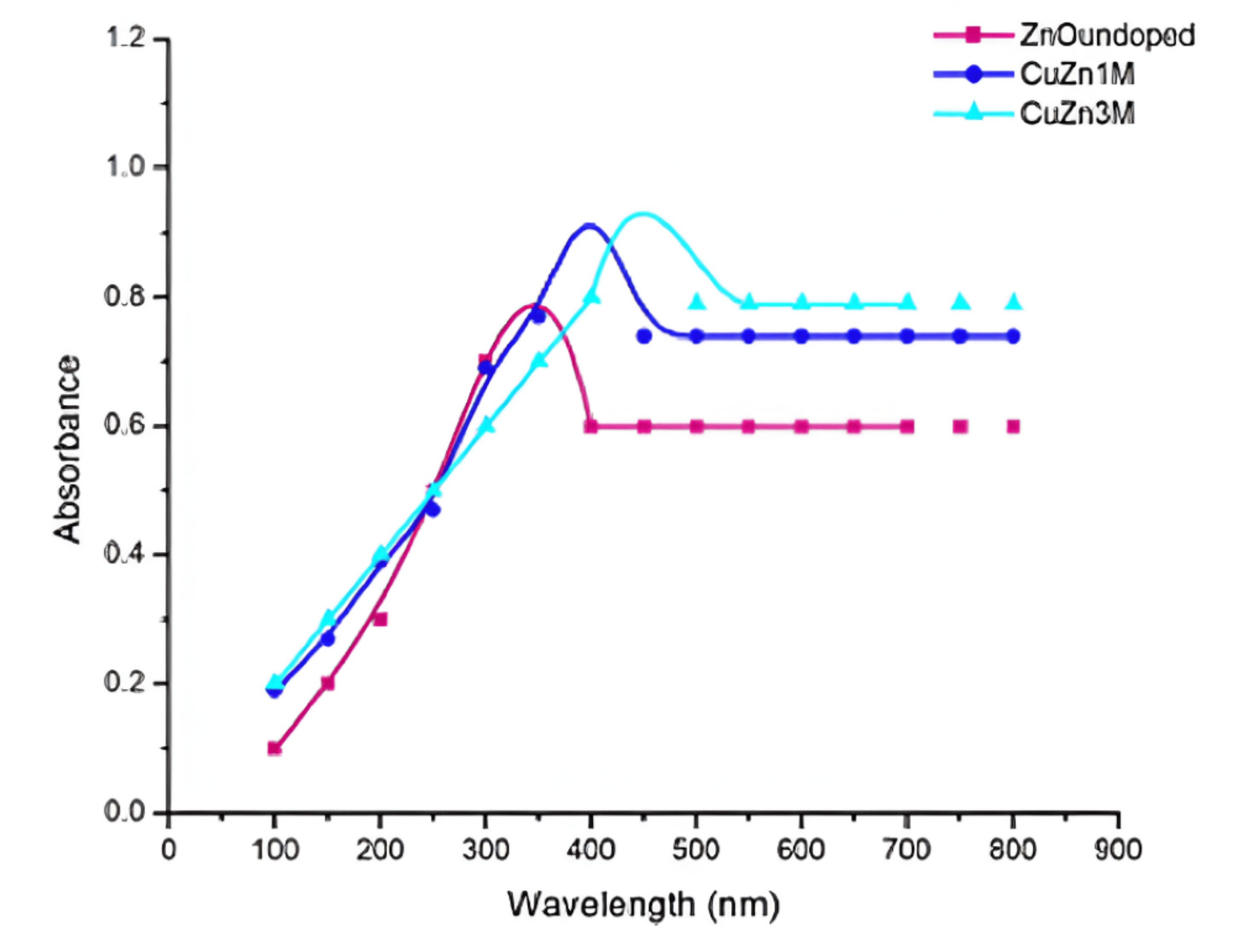
UV-Visible spectra of ZnO and Cu-ZnO NPs demonstrating a red shift with Cu doping. NP, nanoparticle

Figure 5 shows UV-Vis spectra of ZnO and Cu-ZnO NPs. ZnO NPs showed maximum absorption at a wavelength of 370-375 nm. With the introduction of Cu into ZnO, substituting Zn sites with Cu ions, there is a red shift in the absorption edge to longer wavelengths [18].

Scanning Electron Microscopy

**Figure 6:**
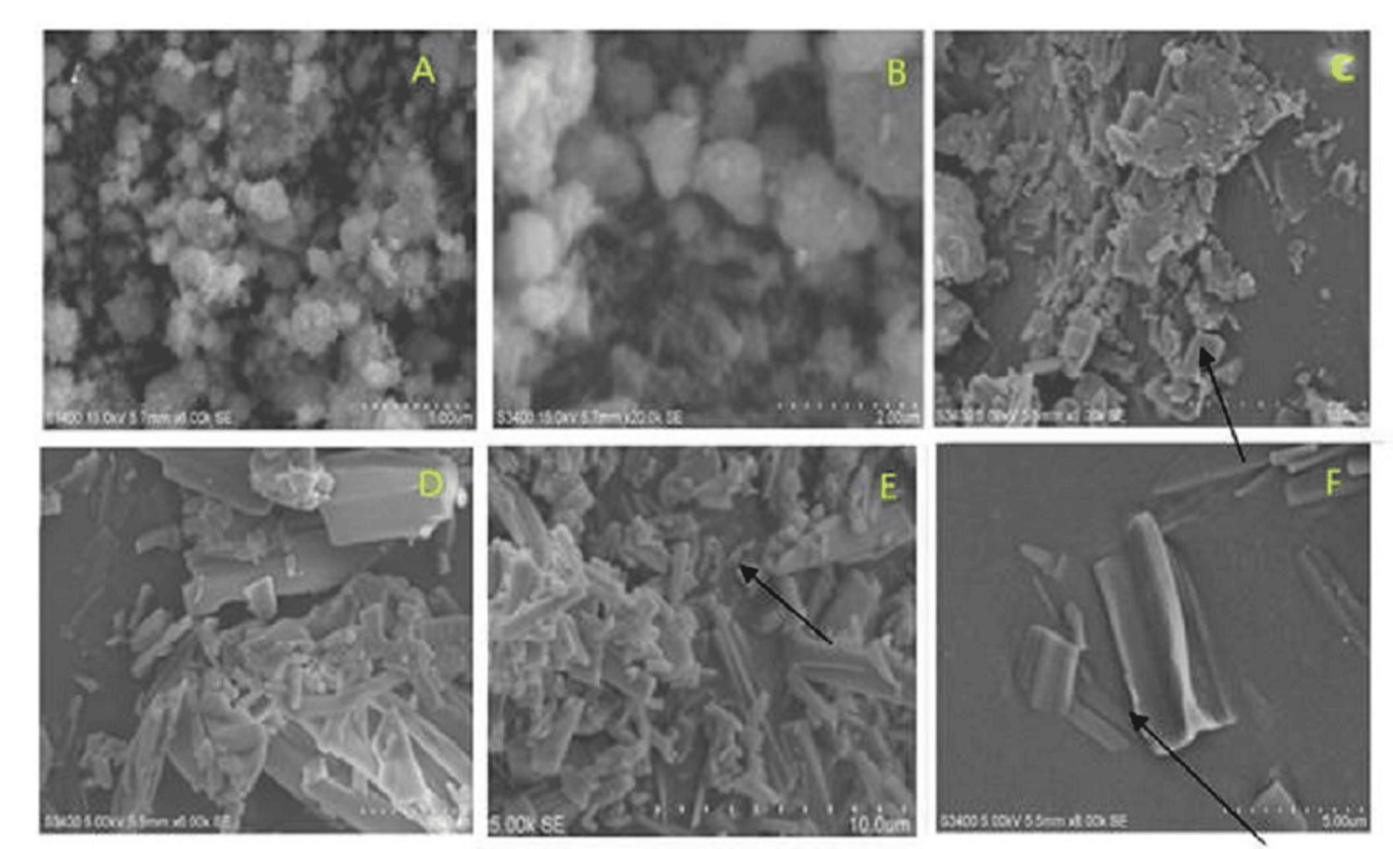
SEM images of ZnO and Cu-ZnO NPs showing definitive morphology and dispersion with an increase in Cu-dopant concentration. ZnO NPs appear as flakes and plates (A and B). Flakes and rod-like appearing 1 M Cu-ZnO NPs (C and D). Short rod-to-tripod-like appearing 3 M Cu-ZnO NPs (E and F). SEM, scanning electron microscopy; NP, nanoparticle

Figures 6A-6F show SEM images of ZnO and Cu-ZnO NPs at various magnifications. ZnO NPs (Figures 6A-6B) appeared irregular and moderately dispersed. With 1 M Cu doping (Figures 6C-6D), the morphology transformed into plate-like to rod-like structures, while 3 M Cu-ZnO (Figures 6E-6F) showed densely packed rod-to-tripod formations. This morphological evolution suggests that Cu²⁺ incorporation into the ZnO lattice enhances nucleation and growth rates, resulting in reduced particle size and altered morphology [19].

Particle Size Determination 

**Figure 7:**
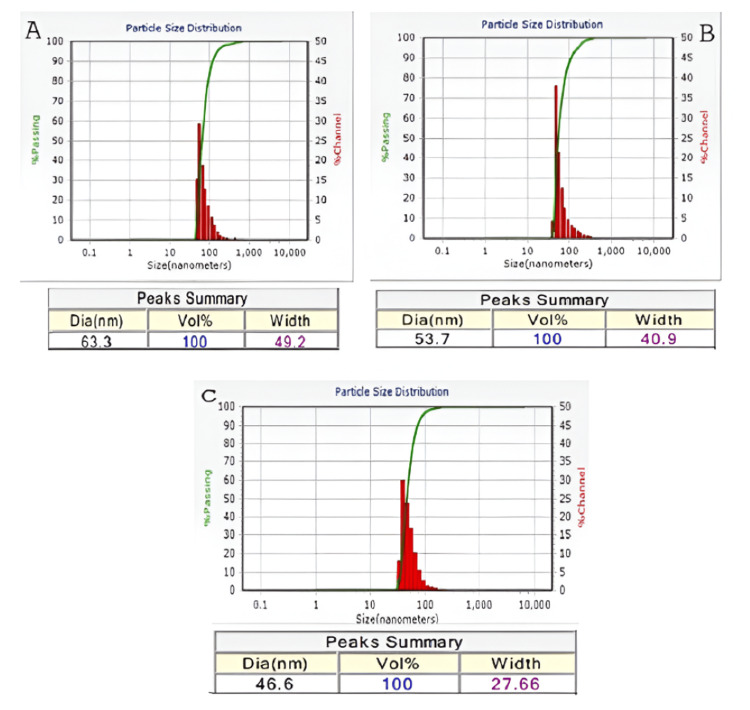
Average particle size of ZnO and Cu-ZnO (1 and 3 M) as determined by DLS. (A) ZnO, (B) Cu-ZnO 1 M, and (C) Cu-ZnO 3 M. DLS, dynamic light scattering

Figures 7A-7C illustrate the average particle size distribution of ZnO and Cu-ZnO NPs. ZnO NPs exhibited an average size of ~63 nm, with a particle size range of 67-68 nm. Upon Cu doping, a clear reduction in size was observed; 1 M Cu-ZnO NPs averaged ~54 nm, while 3 M Cu-ZnO NPs further decreased to ~47 nm with a width of 28 nm, suggesting the formation of rod-like structures. 

Determination of antimicrobial activity of ZnO and Cu-ZnO NPs

**Figure 8:**
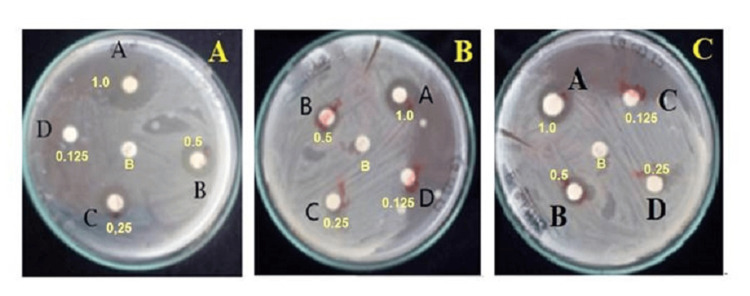
(A) Antimicrobial activity of 3 M Cu-ZnO nanoparticles (NPs) showing a large zone of inhibition; (B) antimicrobial activity of 1 M Cu-ZnO NPs showing a moderate zone of inhibition; (C) antimicrobial activity of ZnO NPs.

In this study, ZnO NPs doped with Cu at two different molar concentrations were evaluated for their antimicrobial activity against *S. aureus*, a common pathogen that causes wound infections, across a concentration range of 1.0-0.125 mg/mL. Table 2 summarizes the average ZOI caused by various concentrations of ZnO and Cu-ZnO NPs (average ZOI calculated using SPSS). Cu-ZnO NPs exhibited enhanced antimicrobial efficacy compared to ZnO, with higher activity observed at increased dopant concentrations. Disk diffusion assay images in Figures 8A-8C further illustrate the superior inhibition of bacterial growth by Cu-ZnO NPs relative to ZnO.

**Table 2:** Antimicrobial activity of ZnO and Cu-ZnO against S. aureus.

ZOI (in mm)	1.0 mg/mL	0.5 mg/mL	0.25 mg/mL	0.125 mg/mL
ZnO	8.5	6.0	0	0
1 M Cu-ZnO	10	5	0	0
3 M Cu-ZnO	14	8.4	5	0

Determination of hematotoxicity of ZnO and Cu-ZnO NPs

**Figure 9:**
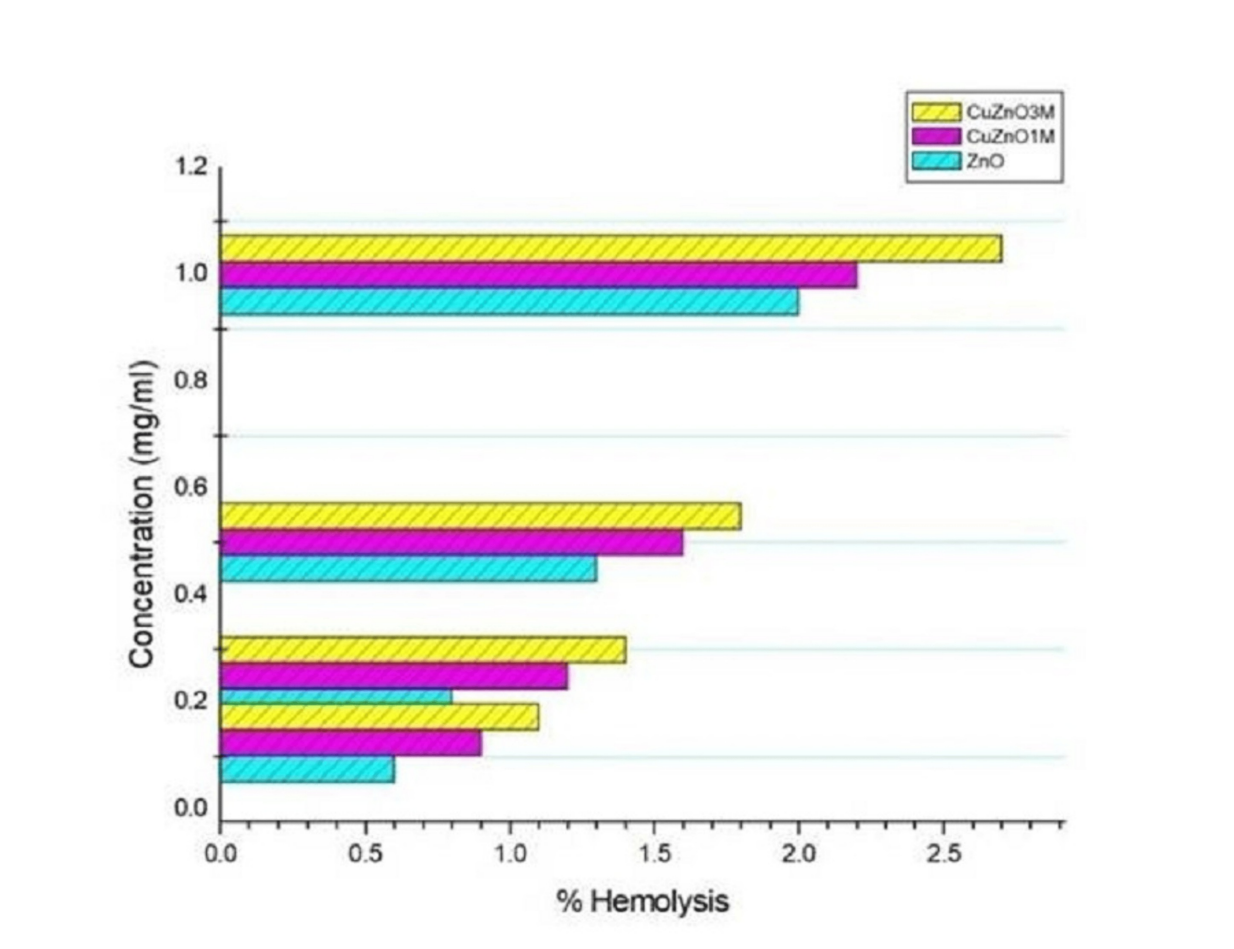
Hemolysis assay results of ZnO and Cu-ZnO NPs. NP, nanoparticle

The hemolysis assay is performed as an initial check to assess possible hematological toxicity toward RBCs, particularly in response to changes in isotonicity caused by drugs, natural compounds, NPs, etc. In this study, hemolysis caused by ZnO NPs and Cu-ZnO NPs was assayed in a concentration range of 1.0-0.125 mg/mL. Among them, 3 M Cu-ZnO exhibited slightly higher hemolysis than 1 M Cu-ZnO NPs; however, both concentrations remained below the accepted toxicity threshold of 5%, indicating minimal disruption of the RBC membrane (Figure 9) [20].

Antifibrotic activity using cell proliferation assay and mitochondrial membrane potential

**Figure 10:**
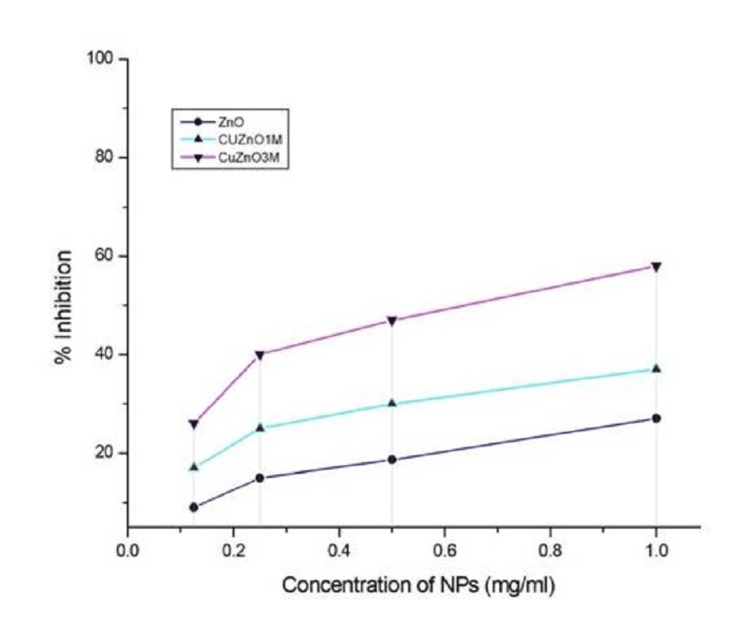
Percentage fibroblast cell inhibition caused by ZnO and Cu-ZnO NPs, showing the effectiveness of antifibrotic activity. NP, nanoparticle

Antifibrotic activity of ZnO and Cu-ZnO NPs was evaluated against Balb 3T3 mouse fibroblast cell lines using WST-8 assay (concentration 1.0 - 0.125 mg/mL), a sensitive colorimetric assay that measures cell viability based on reduction of water-soluble tetrazolium salt to soluble formazan product by mitochondrial NADH in viable cells [15]. The experiments were performed in triplicate, and the average of test results indicated a dose-dependent and molar ratio (Cu) dependent fibroblast inhibition, with Cu-ZnO NPs exhibiting higher inhibitory potential compared to ZnO NPs (Figure 10).

**Figure 11:**
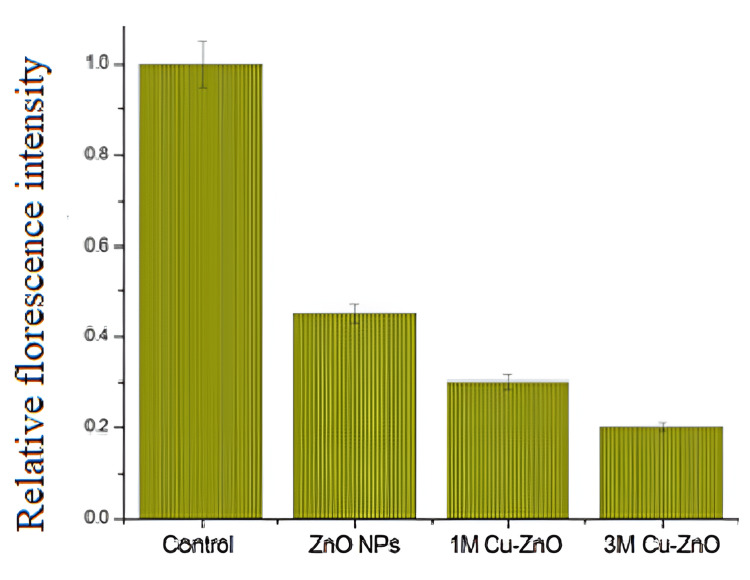
Mitochondrial membrane potential of ZnO and Cu-ZnO NP-treated fibroblast cell lines. NP, nanoparticle

MMP analysis in 3T3 fibroblasts treated with ZnO and Cu-ZnO NPs (1 and 3 M) revealed their impact on mitochondrial health. ZnO NPs maintained stable MMP, indicating healthy mitochondria, while only a slight reduction was observed, suggesting minimal stress (Figure 11) [21]. Treatment with 1 M Cu-ZnO NPs caused a moderate MMP decrease, reflecting mild mitochondrial dysfunction likely due to increased ROS. Among all treatments, the 3 M Cu-ZnO NPs induced the most pronounced MMP loss, indicating severe mitochondrial depolarization and potential apoptosis or necrosis from excessive ROS-mediated damage [16].

## Discussion

Wound healing is a complex phenomenon requiring well-regulated fibroblastic activity for controlled ECM production to aid in the regeneration of scar-free tissue. When this equilibrium is disrupted, excessive fibroblast proliferation and collagen deposition occur, leading to abnormal wound healing outcomes [22]. Therefore, the present-day therapeutic interventions need to focus on the regulation of fibroblastic activity and the prevention of uncontrolled ECM production for balanced wound repair and functional tissue regeneration. Currently available antifibrotic management strategies rely on pharmacological intervention of profibrotic pathways and surgical correction in advanced cases; however, limited localized efficacy and side effects highlight the need to explore locally active alternative therapies.

In the present work, Cu-ZnO NPs were synthesized using *O. sanctum* hydroalcoholic extract, which resulted in highly crystalline wurtzite nanostructures as demonstrated by XRD results. The rising XRD peak intensities at higher Cu ratios confirmed substitutional doping and formation of smaller and ordered crystallites, as substantiated by DLS results [23]. UV-Vis spectroscopy demonstrated a red shift in absorption edge with incorporation of Cu²⁺, indicating narrowing of band gap, enhanced electron-hole separation, and promotion of ROS generation, which also results in enhanced antimicrobial activity and modulation of fibrosis and apoptosis-related signaling pathways [24]. The *O. sanctum* extract, which is used as a bioreductant and capping agent in the present work, is rich in flavonoids, terpenoids, and phenolic compounds and has acted as a natural reducing, capping, and stabilizing agent, leading to eco-friendly synthesis, prevention of NPs agglomeration, and enhanced antimicrobial activity [25]. The 3 M Cu-ZnO NPs showed higher ZOI against *S. aureus* as compared to ZnO and 1 M Cu-ZnO, indicating dose-dependent antimicrobial activity. Cu is a redox-active metal and the first antimicrobial recognized by the U.S. Environmental Protection Agency (EPA). It enhances the antimicrobial and antifibrotic activity of ZnO by generating ROS, releasing cytotoxic Zn²⁺/Cu²⁺ ions, and inhibiting bacterial adhesion through MurA enzyme suppression and biofilm gene downregulation [26].

To evaluate the hematological toxicity of NPs, a hemolysis assay is commonly performed. Hemolysis is the premature destruction of the RBC membrane by various substances entering the body. It leads to the release of cytoplasmic components of RBC, including hemoglobin, decreasing the O_2_ carrying capacity of blood, and leading to conditions like anemia, hypoxia, jaundice, etc. [27]. Our test results showed that both ZnO and Cu-ZnO NPs induced less than 5% hemolysis at the highest tested concentration (1.0 mg/mL), confirming their hemocompatibility within the study range, which indicates that Cu-ZnO NPs, especially at 1 M concentration, are safe for biological applications after further in vitro and in vivo studies.

A dose and dopant ratio-dependent inhibition of 3T3 fibroblast cell proliferation was observed with Cu-ZnO NPs, showing greater inhibition of fibroblast proliferation. These inhibitory effects are directly linked to increased ROS production with enhanced signal triggering, elevated mitochondrial stress, and apoptosis in hyperproliferative fibroblasts [28], which was further confirmed by the determination of MMP activity. The results showed that the 3 M Cu-ZnO group exhibited a significant loss of MMP, reflecting pronounced mitochondrial depolarization due to excessive ROS generation, ultimately leading to apoptotic cell death [29].

The current work provides promising preliminary in vitro antimicrobial and antifibrotic potential of Cu-ZnO NPs synthesized by a green synthesis method. The results of in vitro studies are promising; further in vivo investigations are required to validate the biological safety and efficacy of these green-synthesized ZnO and Cu-ZnO NPs in a complex wound environment. Further complete mechanistic insights will help us better understand the molecular pathways involved in fibroblast modulation. Additionally, future work may focus on assessing long-term biocompatibility and optimizing the dopant ratio for enhanced therapeutic outcomes in the vicinity of the wound environment.

## Conclusions

From the results of this study, it can be concluded that Cu doping effectively modulates the physicochemical attributes of ZnO NPs, leading to enhanced redox potential, antimicrobial efficacy, and antifibrotic activity. Among the tested formulations, 1 M Cu-ZnO NPs demonstrated an optimal balance between antimicrobial potency, antifibrotic effect, and cytocompatibility as seen from the results. These findings highlight the potential of Cu-ZnO NPs as multifunctional agents for wound healing and fibrosis/scar tissue management. Future studies will be focused on in vivo validation, development of various types of pharmaceutical formulations, as well as dose-response modeling to ensure safety and translational applicability in the biomedical field.
